# Exploring the “dark matter” of a mammalian proteome by protein structure and function modeling

**DOI:** 10.1186/1477-5956-11-47

**Published:** 2013-12-09

**Authors:** Michal Brylinski

**Affiliations:** 1 Department of Biological Sciences, Louisiana State University, 70803 Baton Rouge, LA, USA; 2 Center for Computation & Technology, Louisiana State University, 70803 Baton Rouge, LA, USA

## Abstract

**Background:**

A growing body of evidence shows that gene products encoded by short open reading frames play key roles in numerous cellular processes. Yet, they are generally overlooked in genome assembly, escaping annotation because small protein-coding genes are difficult to predict computationally. Consequently, there are still a considerable number of small proteins whose functions are yet to be characterized.

**Results:**

To address this issue, we apply a collection of structural bioinformatics algorithms to infer molecular function of putative small proteins from the mouse proteome. Specifically, we construct 1,743 confident structure models of small proteins, which reveal a significant structural diversity with a noticeably high helical content. A subsequent structure-based function annotation of small protein models exposes 178,745 putative protein-protein interactions with the remaining gene products in the mouse proteome, 1,100 potential binding sites for small organic molecules and 987 metal-binding signatures.

**Conclusions:**

These results strongly indicate that many small proteins adopt three-dimensional structures and are fully functional, playing important roles in transcriptional regulation, cell signaling and metabolism. Data collected through this work is freely available to the academic community at http://www.brylinski.org/content/databases to support future studies oriented on elucidating the functions of hypothetical small proteins.

## Background

Systems biology is an emerging field that aims to comprehend complex interactions within biological systems and, consequently, to shed light on their emergent properties [1]. As a systems-level approach, it requires genome-wide biological data, thus it is greatly facilitated by high-throughput experiments, e.g. whole-genome sequencing. The development of next generation sequencing (NGS) enables researchers to reach into almost complete genomes of numerous species [2,3], revealing more and more details on individual organisms functioning as systems. Despite the continuing advances in data production technologies, the assembly and annotation of particularly complex genomes remain challenging. Difficulties of de novo NGS assembly arise from e.g. contaminating sequences [4], low-quality reads [5], segmental duplications and large common repeats [6]. Another salient flaw is a short-length discontinuity, which has been noted for several assembled genomes [7,8]. Although a substantial fraction of short open reading frames are not genes, many of them have been suggested to encode fully functional proteins [9]. A comparison of the distribution of protein coding sequences from the FANTOM collection of mouse cDNAs [10] against manually curated Swiss-Prot protein database [11] revealed a clear under-prediction of proteins less than 100 residues [12]. The same study estimated that proteins <100aa constitute a 3-fold greater fraction of a mammalian proteome than previously anticipated and provided a solid evidence that the missing small proteins, referred to as a genomic “dark matter”, are in fact functional, often performing novel types of biological function. A recent review examined the growing evidence on the participation of short proteins in numerous cellular processes in bacteria [13]. Several highlighted biological functions include engaging in regulatory processes [14], interacting with a lipid membrane [15] or even modulating its features, acting as chaperones of nucleic acids and metals [16], and stabilizing the structures of larger protein assemblies [17].

As might be expected, a growing interest in small proteins motivates large-scale bioinformatics studies on their molecular functions. For example, small proteins from the mouse proteome were functionally annotated using Pfam database [12]. Another study [18] classified putative genes encoding small proteins across legume genomes according to Gene Ontology [19]. Furthermore, a hierarchical computational approach was proposed to scan a large collection of small protein candidates in *Populus deltoides* leaf transcriptome [20] against known protein domains using InterProScan [21]. Interestingly, by applying sequential filtering by coding potential, interspecies conservation, and protein sequence clustering, known protein domains were identified in 87% of the small protein candidate set. Finally, an analysis using BLAST [22] of the *Drosophila* genome, which is considered as one of the most comprehensively annotated, revealed the existence of at least 401 novel functional small open reading frames [23]. An additional validation of these results by inspecting previously annotated small coding sequences indicated that this number is actually underestimated and there may be as many as 4,561 such functional sequences in *Drosophila*. Bioinformatics techniques to investigate whether putative sequences are actually transcribed include homology-based searches against known protein domains as well as calculating a ratio of non-synonymous to synonymous substitutions indicating protein sequence conservation. A common feature of previously undertaken studies is that purely sequence-based methods have been used; significantly fewer approaches tackle this problem by employing structure-based techniques.

Most computational function-prediction methods rely on inferring relationships between proteins and transfer functional annotations between them [24,25]. One group of annotation approaches widely employ sequence homology-based inference under the assumption that a common origin of homologues is reflected in their structure and function [26,27]. Nevertheless, homology-based transfer is complicated by many factors, e.g. proteins may acquire new functions as they evolve [28,29]. Consequently, the possibility of chains of misannotation exists [30], causing notably high levels of misannotation across public databases [31]. In that regard, structure-based methods have been developed [32]; for example, many functional aspects of proteins can be effectively transferred from structural neighbors [33]. However, it has been demonstrated that using structure similarity alone may lead to a relatively high false positive rate in protein function annotation [34]. Moreover, structure-based methods typically require high-quality target structures, preferably solved by X-ray crystallography or NMR, which considerably hinders their application in large-scale annotation efforts. More recently, evolution/structure-based approaches to protein function inference have emerged to address the limitations of purely sequence- and structure-based methods [35]. These powerful techniques effectively combine both sequence and structure components and cover many aspects of protein molecular function [36]. From a point of view of across-genome function annotation, an important feature of evolution/structure-based approaches is their remarkably high tolerance to distortions in target structures, thus even moderate-quality protein models can be included in the modeling process. Accordingly, using these techniques maximizes the coverage of targeted gene products concurrently maintaining a high accuracy of function prediction.

In this study, we describe the application of a collection of evolution/structure-based algorithms to perform structural and functional characterization of small proteins, referred to as sproteins, identified in the mouse proteome. First, we construct their structure models, which are subsequently subject to structure classification using CATH Protein Structure Classification Database [37]. Structure studies are followed by comprehensive function annotation considering a number of functional aspects including interactions with small organic molecules, e.g. metabolites, other proteins as well as metal ions. The results indicate that many sproteins adopt well-defined three-dimensional structures and perform important molecular functions. These findings should provide useful guidance for the design of future experiments.

## Results and discussion

### 3D structures can be modeled for nearly half of small proteins

**Figure 1:**
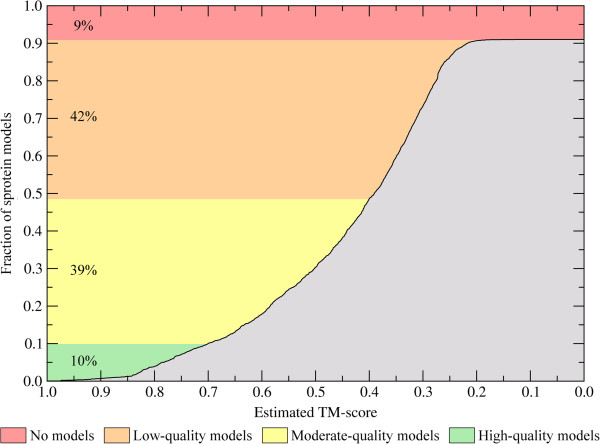
**Confidence of sprotein structure modeling.** The results are presented as a cumulative fraction of target sequences for which the structure was modeled to an estimated TM-score displayed on the *x*-axis. Based on confidence estimates, theoretical sprotein structures are categorized as high-, moderate- and low-quality models.

The first step in our study is the construction of three-dimensional molecular structures for 3,556 sproteins in the mouse proteome. Here, we use *e*Thread, a template-based approach [38,39], which can generate correct structures and provides reliable confidence estimates for modeling accuracy in terms of the expected TM-score [40] to native. Figure 1 shows that high-quality models, whose TM-score estimated by *e*Rank is ≥0.7, are constructed for 10% of the target sequences; for proteins 50–100 residues in length, a TM-score of ≥0.7 corresponds to a median backbone Cα-RMSD of 2.8 Å. For another 39% of sproteins, the estimated TM-score is ≥0.4 indicating moderate structural quality (median Cα-RMSD of 6.4 Å). No confident models with a statistically significant TM-score are generated for 42% of the targets. For these low-quality models, the expected Cα-RMSD is >11 Å, which is a typical value for random structures within this length range [41]. Finally, for 9% of the sequences, meta-threading failed to detect any templates, thus no models are constructed. We also compare the confidence estimates by *e*Rank to these calculated by APOLLO, which is an alternative structure-based quality assessment method [42]. Additional file 1: Figure S1 shows that both confidence values are in good agreement with the Pearson correlation coefficient (CC) of 0.5. Nevertheless, TM-score estimates by *e*Rank are more correlated with the real TM-score values than these by APOLLO [39] (CC is 0.89 and 0.77, respectively); therefore, the former is used in this study as the primary quality assessment method.

**Figure 2:**
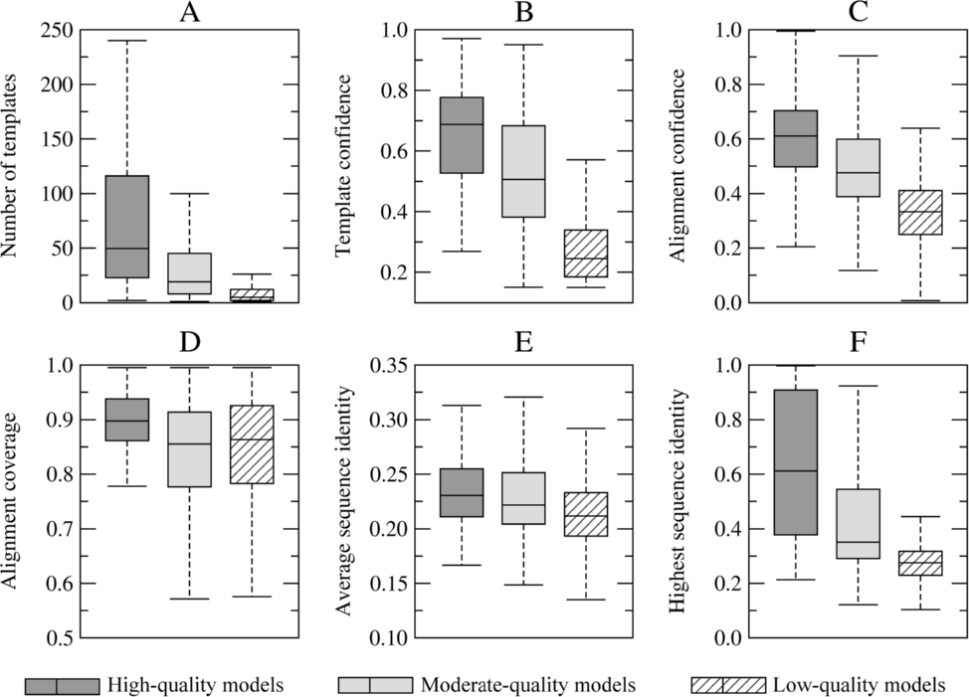
**Threading results for sprotein sequences. (A)** The number of detected templates per target, **(B)** the average confidence for template identification, **(C)** the confidence of target-to-template alignments, **(D)** the coverage of target sequences by threading alignments, **(E)** the average target-template sequence identity, and **(F)** the highest target-template sequence identity. Target proteins are divided into three groups according to the confidence of structure modeling. Boxes end at the quartiles Q_1_ and Q_3_ and whiskers point at the farthest points that are within 3/2 times the interquartile range; a horizontal line in a box is the median.

In template-based protein structure modeling, the quality of a final model is closely coupled to the accuracy and confidence of template identification. In Figure 2, for sprotein models categorized into three groups (high-, moderate- and low-quality models), we analyze the most important statistics reported by meta-threading using *e*Thread. High- (moderate-) quality models typically require multiple templates with a median value of 50 (19), see Figure 2A. Importantly, as shown in Figures 2B and C, the confidence of template selection and alignment construction is also high: the median value is 0.69 (0.51) and 0.61 (0.48), respectively. Figure 2F shows that these estimates are correlated with the sequence identity of the most similar template, which is 61% for high-quality models indicating close evolutionary relationships. For moderate-quality models the median highest target-template sequence identity is 35%; however, the signal detected by profile-profile comparison is still strong enough to generate weakly homologous, yet confident models with an estimated TM-score of ≥0.4. Unreliable sprotein models were constructed using on average only 5 templates, whose selection confidence, alignment confidence and the highest sequence identity to the target is 0.24, 0.33 and 27%, respectively. As shown in Figures 2D and E, the average alignment coverage and the average target-template sequence identity are comparable across the three sets of protein models.

### Most small proteins are mainly helical

**Figure 3:**
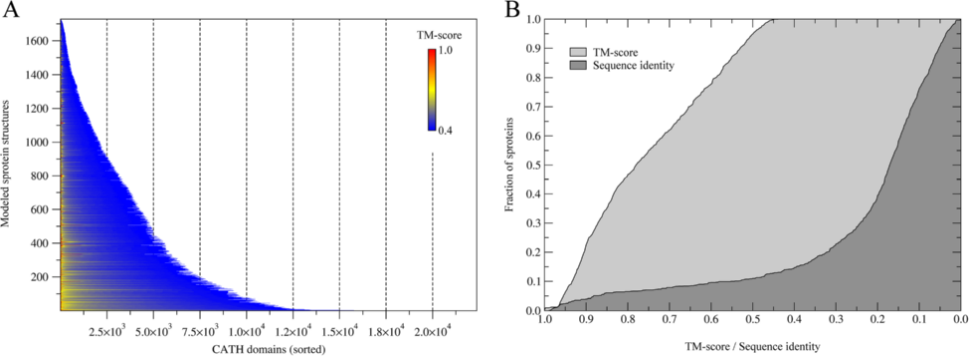
**CATH structural matches for sprotein models. (A)** For each target, the CATH domain hits are sorted according to the TM-score. **(B)** Cumulative distribution of TM-score and sequence identity of the best structural match identified for sprotein models.

Next, we use a nearest-neighbor approach to identify in the CATH library structural matches for confidently modeled sprotein structures. The results of structural alignment calculations are presented in Figure 3. Figure 3A shows that for all models, at least one CATH structure is identified at a TM-score threshold of 0.4. Furthermore, for roughly 900, 400 and 200 sprotein models, as many as 2,500, 5,000 and 7,500 structurally similar domains are found in CATH. Focusing on the closest structural match (Figure 3B), a highly significant CATH match with a TM-score of ≥0.7 (≥0.5) is identified for 62% (95%) of sprotein models. We note that these are structural analogs, which are not necessarily evolutionarily closely related; only 11% of nearest neighbors share at least 50% sequence identity with their sprotein targets.

In addition to the global structure quality, we also assess the local structural features and compare them to these calculated across experimental structures of the closest CATH matches. Table 1 shows that most sproteins are mainly helical, with 40% and 34% of residues assigned to α-helical conformation in high- and moderate-quality models, respectively. This composition is in good agreement with the secondary structure assignment for best CATH matches, which contain a significant fraction of helical residues (42%). β-Structures are modeled with a slightly lower accuracy. 17-19% of residues in equivalent CATH domains are in the extended conformation, whereas in high- and moderate-quality models, 15% and 10% residues are assigned to β-structure, respectively. Consequently, the content of turn residues in sprotein models is higher compared to CATH structures. In general, β-structures are more difficult targets for modeling than α-helices due to non-local interaction patterns. Hydrogen bonding is one of the major criteria in secondary structure assignment; Table 2 shows that significantly less main-main chain hydrogen bonds are formed in the high- and moderate-quality structures than in the corresponding CATH domains (55%, 45% and 61%, respectively). Despite these imperfections in hydrogen bonding pattern, the backbone stereochemical quality in sprotein models is comparable to that in the crystal structures of equivalent CATH domains (Table 3). For high- and moderate-quality models, 89% and 85% residues are assigned by PROCHECK [43] to most favored regions of the Ramachandran space, respectively; this is only 1% and 4% less than in CATH structures, respectively. We note that function annotation protocols applied to the modeled structures of sproteins are fairly insensitive to local (and to some extent global as well) distortions, thus the quality of these models is sufficient for structure-based functional analyses.

**Table 1:** Secondary structure content in sprotein models

**Class**^***a***^	**Modeled sprotein structures**			**Best CATH matches**	
	**High-quality**	**Moderate-quality**	**Low-quality**	**High-quality**	**Moderate-quality**
α-Helix	0.40 ±0.23	0.34 ±0.24	0.34 ±0.23	0.42 ±0.23	0.42 ±0.25
3-10 Helix	0.02 ±0.03	0.02 ±0.03	0.02 ±0.03	0.03 ±0.03	0.04 ±0.03
π-Helix	0.00 ±0.00	0.00 ±0.00	0.00 ±0.00	0.00 ±0.00	0.00 ±0.00
Extended	0.15 ±0.17	0.10 ±0.12	0.05 ±0.09	0.19 ±0.18	0.17 ±0.16
Isolated bridge	0.01 ±0.01	0.01 ±0.01	0.01 ±0.01	0.01 ±0.01	0.01 ±0.01
Turn	0.22 ±0.09	0.29 ±0.13	0.33 ±0.15	0.19 ±0.08	0.19 ±0.09
Coil	0.20 ±0.08	0.25 ±0.11	0.26 ±0.12	0.16 ±0.05	0.17 ±0.07

^*a*^ According to STRIDE classification.

Secondary structure composition of modeled sprotein structures is calculated by STRIDE as a fraction of residues assigned to different secondary structure classes. Sprotein models are compared to a set of the best structural matches identified in the CATH library by Fr-TM-align.

**Table 2:** Hydrogen bond pattern in sprotein models

**Hydrogen bond type**^***a***^	**Modeled sprotein structures**			**Best CATH matches**	
	**High-quality**	**Moderate-quality**	**Low-quality**	**High-quality**	**Moderate-quality**
Main-main chain	0.55 ±0.13	0.45 ±0.19	0.42 ±0.19	0.61 ±0.09	0.61 ±0.14
Side-side chain	0.02 ±0.02	0.02 ±0.02	0.02 ±0.02	0.09 ±0.04	0.09 ±0.04
Main-side chain	0.10 ±0.04	0.10 ±0.04	0.10 ±0.05	0.18 ±0.07	0.17 ±0.06

^*a*^ According to HBPLUS classification.

Number of hydrogen bonds per residue is calculated by HBPLUS for modeled sprotein structures. Sprotein models are compared to a set of the best structural matches identified in the CATH library by Fr-TM-align.

**Table 3:** Stereochemical quality of sprotein models

**Φ/Ψ Region**^***a***^	**Modeled sprotein structures**			**Best CATH matches**	
	**High-quality**	**Moderate-quality**	**Low-quality**	**High-quality**	**Moderate-quality**
Most favored	0.89 ± 0.04	0.85 ± 0.07	0.81 ± 0.09	0.90 ± 0.05	0.89 ± 0.07
Additional allowed	0.08 ± 0.03	0.11 ± 0.05	0.13 ± 0.06	0.09 ± 0.04	0.10 ± 0.06
Generously allowed	0.02 ± 0.01	0.02 ± 0.02	0.03 ± 0.03	0.01 ± 0.01	0.01 ± 0.01
Disallowed	0.01 ± 0.01	0.02 ± 0.02	0.03 ± 0.03	0.00 ± 0.00	0.00 ± 0.00

^*a*^ According to PROCHECK classification.

Stereochemical quality of modeled sprotein structures is calculated by PROCHECK as a fraction of residues assigned to different regions of Ramachandran map. Sprotein models are compared to a set of the best structural matches identified in the CATH library by Fr-TM-align.

**Figure 4:**
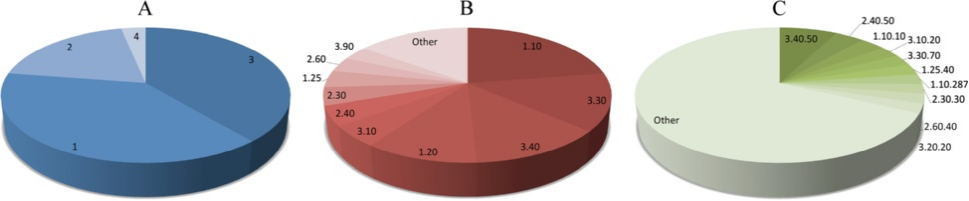
**Structural classification of sprotein models according to CATH. (A, B and C)** Assignment at the class, architecture and topology level, respectively. In **B** and **C**, only ten largest groups are labeled.

**Figure 5:**
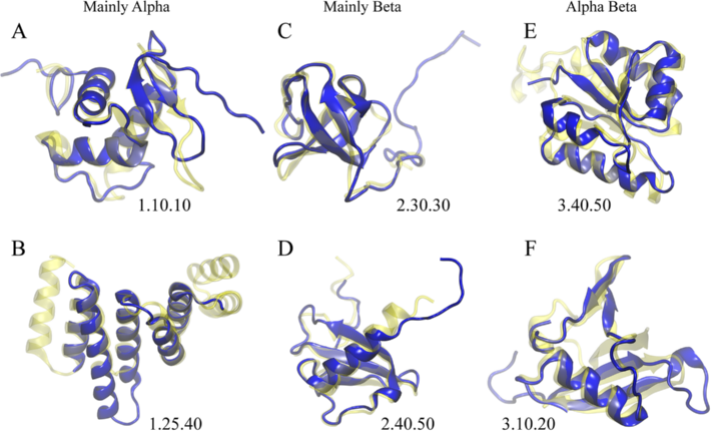
**Examples of CATH domain matches for sproteins.** Two representative examples are selected for each major class: **(A, B)** mainly alpha, **(C, D)** mainly beta, and **(E, F)** alpha beta. The modeled sprotein and the CATH match are shown in solid blue and transparent yellow, respectively, for **(A)** D230048N02/3coaC00, **(B)** 9330209D09/2fo7A00, **(C)** C330006B10/1ng2A02, **(D)** A430052M02/1esrA00, **(E)**, B130011N18/1c30A08, and **(F)** 4832408G11/1h4rA01.

Finally, using structure alignments of sprotein models to the CATH database of domain structures, we approximate the structural classification of sproteins. CATH features four levels of classification: class, architecture, topology and homologous superfamily [37]. The results for class, architecture and topology assignments are shown in Figure 4. At the highest hierarchy level, the majority of sproteins are assigned to Alpha Beta (3, 38.8%) and Mainly Alpha (1, 38.6%) classes, see Figure 4A. Figure 4B shows that in class 3, 13.7% and 12.9% sproteins are assigned 2-Layer Sandwich (3.30) and 3-Layer Sandwich (3.40) architecture, respectively. In class 1, 22.6% and 10.8% sproteins are categorized as Orthogonal Bundle (1.10) and Up-down Bundle (1.20), respectively. The most abundant topologies presented in Figure 4C include Rossman fold (3.40.50, 7.6%), OB fold (2.40.50, 3.8%), Arc Repressor Mutant subunit A (1.10.10, 3.3%), Ubiquitin-like UB roll (3.10.20, 2.8%), and Alpha-Beta Plaits (3.30.70, 2.7%). Two representative examples of sproteins from each major class aligned onto their best CATH matches are shown in Figure 5. On the whole, our structural analysis corroborates earlier studies suggesting that sproteins exhibit significant structural diversity [13].

### Small proteins form protein-protein interactions

**Figure 6:**
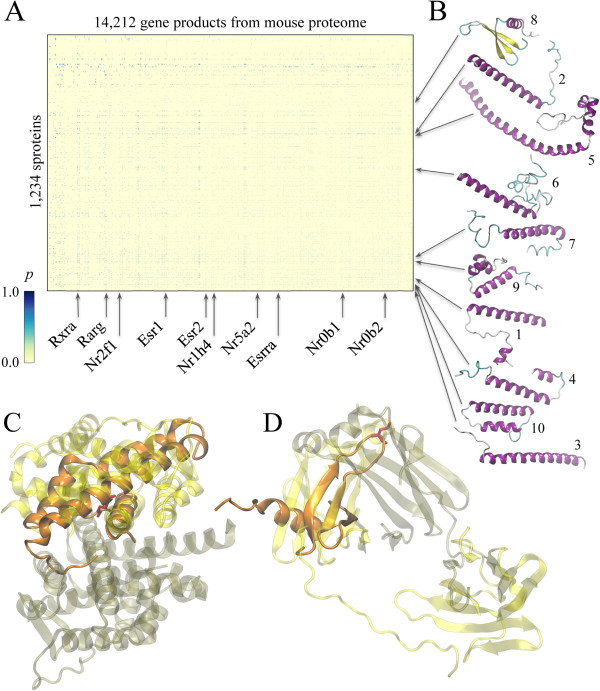
**Putative protein-protein interactions involving sproteins. (A)** A heat map showing the probability of interaction *p* according to the color scale on the left. **(B)** Representative examples of “promiscuous” sproteins, labeled according to Table 4. **(C, D)** Examples of sproteins (solid orange) superposed onto the parental dimeric templates (transparent): **(C)** ligand – D630037N19, receptor – ENSMUSP00000039175, template – estrogen receptor α (PDB-ID: 2qgw); and **(D)** ligand – I830091D09, receptor – ENSMUSP00000122045, template – VpreB protein (PDB-ID: 2h3n). Template receptors and ligands are colored in tan and yellow, respectively. Hot spot residues identified by PINUP are shown as red sticks.

Macromolecular interactions between sproteins and the remaining gene products from the mouse proteome are modeled using a combination of structure alignments, sequence profile-profile comparisons, an empirical scoring function for binding residue prediction and statistical protein docking potentials. Here, we consider 1,234 sprotein targets for which high- and moderate-quality structural models are constructed, and 14,212 mouse gene products that can be confidently mapped to the known crystal structures of receptor proteins using profile HMM-HMM alignments. Figure 6A shows the heat map of putative protein-protein interactions; out of >1.7 × 10^7^ theoretical interactions, 178,745 are assigned a probability of ≥0.5 by an energy-based approach calibrated on the crystal structures of protein-protein complexes (see Additional file 2: Figure S2). Putative assemblies involving sproteins presented in Figures 6C and D are examples of α-helical and β-structure interfaces, respectively. The first complex between D630037N19 and Nr0b2 was modeled based on the steroid-binding region of estrogen receptor α (PDB-ID: 2qgw) and has favorable interaction energy of -0.67, which corresponds to an interaction probability of 0.75. For the second complex between I830091D09 and immunoglobulin lambda-like polypeptide 1, constructed using the crystal structure of VpreB protein (PDB-ID: 2h3n), interaction energy and the corresponding probability is -0.39 and 0.65, respectively. Note that in both cases, hot spot residues identified in sproteins by PINUP [44] (red sticks in Figures 6C and D) are correctly located within the putative protein-protein interface.

Arrows in Figure 6 point at the most “promiscuous” sproteins and receptors (rows and columns of the heat map, respectively) involved in multiple protein-protein interactions. These are further summarized in Tables 4 and 5. For example, several sproteins that belong to Ferritin, Fumarase C, Hemaggutinin ectodomain and Helix hairpins topologies are predicted to interact with >1,500 receptor proteins (Table 4). As shown in Figure 6B, a common feature of these proteins is a high helical content. Studies focusing on protein interfaces reveal that α-helices located on protein surface form bioactive regions responsible for the recognition of other macromolecules, thus often mediate protein-protein interactions [45,46]. Table 5 lists the most “promiscuous” receptors from mouse proteome predicted to form interactions with sproteins. Interestingly, many of these proteins belong to nuclear receptor family of signal-regulated transcription factors that play a critical role in development and homeostasis of multicellular organisms [47,48]. A special feature of nuclear receptors is their ability to recruit a significant number of other proteins to facilitate the process of gene transcription [49,50]. Our large-scale modeling of putative protein-protein interactions suggests that many uncharacterized sproteins may act as upstream target proteins directly linked to transcription inhibitory mechanisms in mammalian cells. This is also consistent with previous findings suggesting that many sproteins localize to perinuclear space and play roles in cell signaling [12].

**Table 4:** Examples of protein-protein interactions involving sproteins

**Rank**	**Sprotein**	**Model confidence**^***a***^	**PPI**^***b***^	**CATH assignment**		
				**Domain**	**TM-score**^***c***^	**Classification**
1	B930036P11	0.48	1,757	1ji4A00	0.82	1.20.1260 (Ferritin)
2	E430007D20	0.47	1,638	2x75A02	0.59	1.20.200 (Fumarase C)
3	G530013D06	0.55	1,596	3m5jB00	0.80	3.90.20 (Hemagglutinin ectodomain)
4	A730094F08	0.44	1,525	1pd3A00	0.75	1.10.287 (Helix hairpins)
5	1110020 M21	0.46	1,420	1wp1B01	0.57	1.20.1600 (Outer membrane efflux proteins)
6	2310075O16	0.45	1,416	3ud0A00	0.56	1.10.3080 (Clc chloride channel)
7	6720468P07	0.41	1,390	1wdzA00	0.62	1.20.1270 (Substrate binding domain of Dnak)
8	I830091D09	0.83	1,311	1icwB00	0.80	2.40.50 (Dihydrolipoamide Acetyltransferase)
9	G630033A22	0.52	1,295	1y9qA01	0.71	1.10.260 (434 Repressor, N-term)
10	K430331D04	0.41	1,287	2jexA01	0.97	1.10.287 (Helix hairpins)

^*a*^ TM-score for the top structural model estimated by *e*Thread; ^*b*^ number of putative protein-protein interactions with the remaining gene products in the mouse proteome; ^*c*^ TM-score between the structural model and the top CATH domain hit.

Ten most “promiscuous” sproteins and their CATH assignment.

**Table 5:** Examples of protein-protein interactions involving sproteins

**Rank**	**Receptor ensembl ID**	**PPI**^***a***^	**UniProt**		
			**ID**	**Name**	**Description**
1	ENSMUSP00000039175	118	Q62227	Nr0b2	Nuclear receptor subfamily 0 group B member 2
2	ENSMUSP00000118161	117	B8JJI9	Nr2f1	Nuclear receptor subfamily 2, group F, member 1
3	ENSMUSP00000025906	116	O08580	Esrra	Steroid hormone receptor ERR1
4	ENSMUSP00000101214	115	P19785	Esr1	Estrogen receptor
5	ENSMUSP00000067266	115	P18911	Rarg	Retinoic acid receptor gamma
6	ENSMUSP00000076491	114	P28700	Rxva	Retinoic acid receptor RXR-alpha
7	ENSMUSP00000106051	114	O08537	Esr2	Estrogen receptor beta
8	ENSMUSP00000027649	113	P45448	Nr5a2	Nuclear receptor subfamily 5 group A member 2
9	ENSMUSP00000053092	108	Q60641	Nr1h4	Bile acid receptor
10	ENSMUSP00000026036	107	Q61066	Nr0b1	Nuclear receptor subfamily 0 group B member 1

^*a*^ Number of putative interactions with sproteins.

Ten most “promiscuous” receptors forming putative interactions with sproteins.

### Small proteins interact with ligands

**Figure 7:**
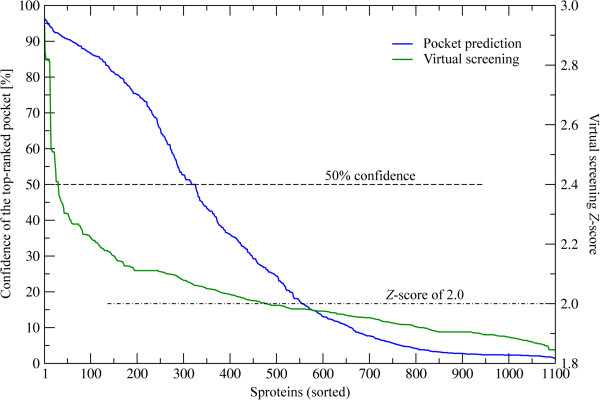
**Confidence of ligand binding prediction for sproteins.** Two confidence estimates are plotted: for the location of the top-ranked binding site (blue, left *y*-axis) and for the reliability of ligand virtual screening (green, right *y*-axis). The horizontal dashed and dot-dash lines show 50% confidence and 2.0 *Z*-score thresholds, respectively.

**Figure 8:**
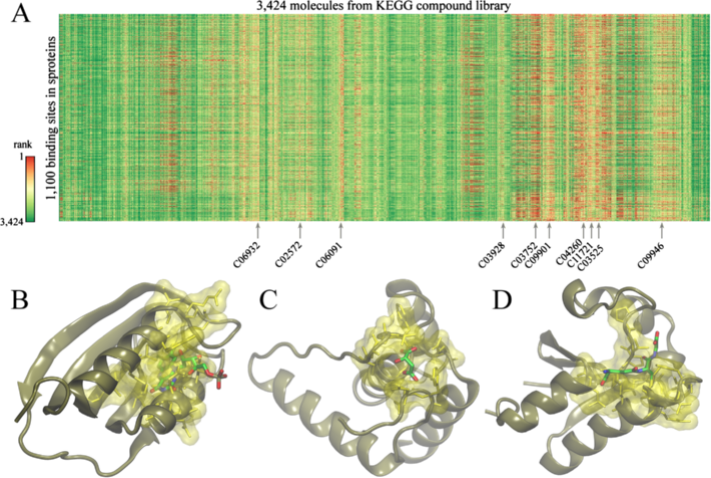
**Virtual screening against sproteins. (A)** Putative interactions between sproteins and a non-redundant subset of KEGG compound library. Color scale shows the rank (scaled to log_2_) assigned to a KEGG compound by virtual screening against a putative binding site in sprotein. **(B, C and D)** Examples of highly confident ligand binding site predictions for sproteins 1700008E22, 1300005 N15 and 4833429C11, respectively. Predicted binding residues are colored in yellow (solid sticks and transparent surfaces). Binding ligands (flavin mononucleotide, D-malate and glutathione, respectively) transferred from template proteins (BLUF photoreceptor, phosphatase 23 and glutaredoxin S12, respectively) are shown as solid sticks colored by atom type.

**Figure 9:**
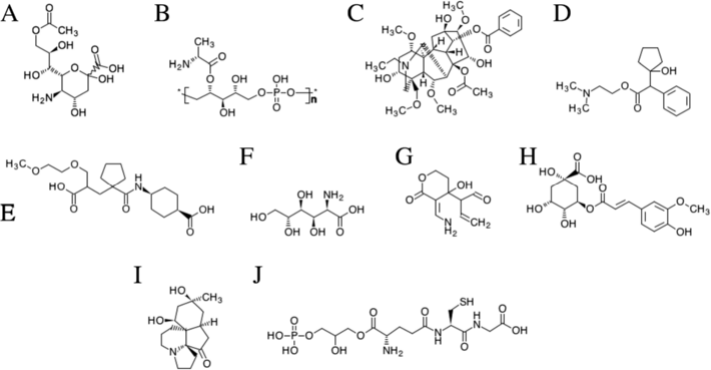
**Examples of ligands binding to sproteins.** Ten top-ranked KEGG compounds selected by virtual screening against sproteins (KEGG-ID and the average *Z*-score from virtual screening against 1,100 binding sites in sproteins are given in parentheses): **(A)** O-acetylneuraminic acid (C03525, 1.042), **(B)** O-D-alanyl-poly(ribitol phosphate) (C04260, 1.014), **(C)** aconitine (C06091, 0.952), **(D)** cyclopentolate (C06932, 0.950), **(E)** candoxatrilat (C11721, 0.944), **(F)** D-glucosaminate (C03752, 0.902), **(G)** enicoflavine (C09946, 0.854), **(H)** 5-O-feruloylquinic acid (C02572, 0.812), **(I)** serratine (C09901, 0.805), and **(J)** 3-phosphoglycerol-glutathione (C03928, 0.761).

Evolution/structure-based approaches are state-of-the-art modeling techniques widely used in ligand binding prediction. A unique feature of these methods is their applicability not only to experimentally solved structures, but also to theoretical models. Using *e*FindSite [51], we identified putative ligand binding sites in 1,100 sproteins with confidently modeled structures. Importantly, *e*FindSite offers a reliable system for estimating the prediction accuracy. As shown in Figure 7, ligand binding regions are predicted with a high (≥50%) confidence for 325 sproteins. In addition, each putative binding site was subject to virtual screening against the KEGG compound library [52] to identify potential binders. The confidence of ligand ranking is expressed by a *Z*-score of the top-ranked compound; *Z*-score values of ≥2 typically indicate reliable predictions. Figure 7 shows that putative binding ligands are confidently predicted for 478 sproteins. KEGG compound library comprises a large collection of small molecules that bind to proteins; we can identify these compounds that bind to multiple sproteins. The results of this analysis are presented in Figure 8A as an all-against-all matrix with ligand ranks shown in color scale. Arrows indicate the locations of ten top-ranked KEGG compounds, which are also presented in Figure 9. These include several metabolites, such as amino carbohydrates O-acetylneuraminic acid and D-glucosaminate, which confirm that sproteins play roles in metabolism [12]. Natural product alkaloids aconitine, enicoflavine and serratine identified in our analysis as binders to sproteins accord with their reported roles in pathogen protection [53]. Other examples of the top-ranked KEGG compounds include pharmacological agents cyclopentolate and candoxatrilat, as well as a glutathione derivative, 3-phosphoglycerol-glutathione. Importantly, our structure-based approach also allows investigating protein-ligand interactions at the molecular level. Figures 8B-D show representative examples of ligand binding sites predicted in sprotein models depicting putative interactions with flavin mononucleotide, D-malate and glutathione. These results may provide useful guidance for the design of experiments focusing on small molecule binding to sproteins.

### Small proteins bind metal ions

**Figure 10:**
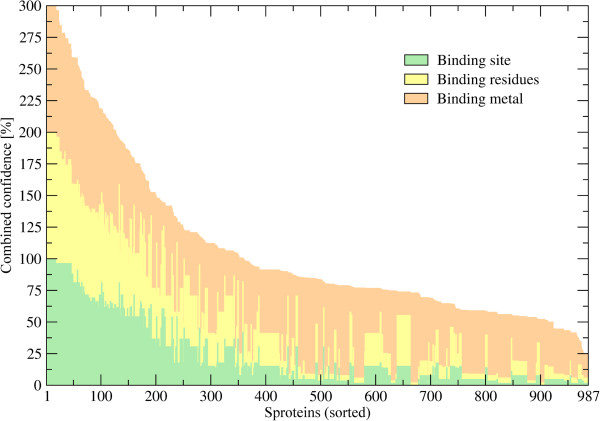
**Confidence of metal binding prediction for sproteins.** FINDSITE-metal provides three confidence estimates for: metal-binding sites, residues and the type of binding metal; these can add up to a combined confidence of 300% and are shown on the *y*-axis. 987 sproteins annotated by FINDSITE-metal shown on the *x*-axis are sorted according to the combined confidence.

**Figure 11:**
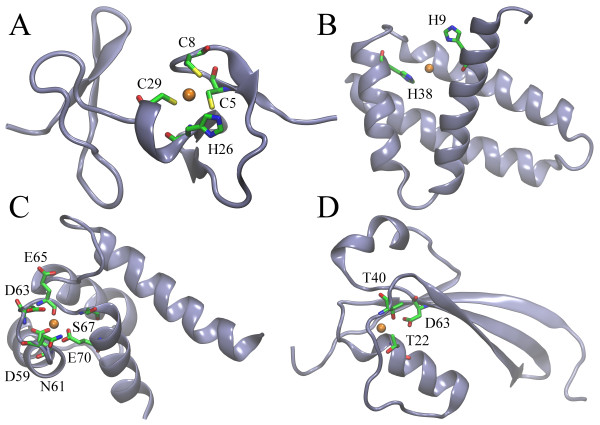
**Representative examples of metal binding sites in sproteins. (A)** Zinc-binding site in 9630046D09, **(B)** iron-binding site in 2810007 M22, **(C)** calcium binding site in I920026J24, and **(D)** magnesium-binding site in I420022F17. Putative positions of binding metals and predicted binding residues are shown as orange balls and sticks colored by atom type, respectively.

Finally, using FINDSITE-metal [54], we detect putative metal binding sites across a set of confidently modeled sprotein structures. At least one metal binding site was predicted for 987 proteins. FINDSITE-metal offers three separate confidence estimates for the prediction of binding site location, binding residues as well as the class of binding metal. Note that this system was rigorously calibrated against a large dataset of metal binding proteins [54]. Figure 10 shows that confident predictions are obtained for a significant fraction of putative metal binding sproteins. Specifically, 19.1%, 20.7% and 72.5% of sproteins are assigned a high confidence of ≥50% with respect to the prediction of site location, binding residues and the type of binding metal, respectively. Furthermore, the most abundant classes of binding metal include calcium, zinc and magnesium, which are predicted to form complexes with 29.8%, 29.3% and 24.1% of putative metallo-sproteins. Nickel, iron, copper, manganese and cobalt are assigned to 5.7%, 4.3%, 2.7%, 2.2% and 1.9% of the targets, respectively. This composition of the metal binding complement identified by FINDSITE-metal across a set of sproteins from the mouse proteome is in good qualitative agreement with proteome-wide estimates collected for other organisms [55,56]. It is important to point out that many metal binding sites in proteins are non-local in sequence without any distinct spacing patterns [57,58], therefore are undetectable using simple sequence-based approaches. Here, structure-based methods generally provide a higher coverage. This is illustrated in Figure 11, which features several representative examples of confidently predicted sites in sprotein models that bind to zinc, iron, calcium and magnesium (Figures 11A, B, C and D, respectively). Our approach not only effectively recognizes the distinctive geometrical features of metal binding sites in protein models, but also accounts for the identity of binding residues to ensure that the predicted locations provide a proper chemical environment for binding of different metals. Although sproteins are rather unlikely to perform enzymatic reactions by themselves, they may function as metal chaperones [13]. For instance, MntS gene in *Escherichia coli* was found to encode a small, 42 amino acid in length, sprotein, which is hypothesized to facilitate the association with manganese of another protein, MntR [16].

## Conclusions

In this study, we apply a collection of tools for evolution/structure-based function annotation of small proteins identified in the mouse proteome. Our results indicate that many of these putative proteins adopt a well-defined tertiary structure with 95% of sprotein models confidently matched to known proteins from the CATH database. Structure modeling reveals that the majority of sproteins are characterized by a relatively high helical content and belong to α/β and mainly α classes. Function-oriented modeling of protein-protein interactions suggests that many sproteins are involved in transcriptional regulation and cell signaling. Furthermore, large-scale virtual screening simulations indicate that sproteins have capabilities to bind a wide range of small organic compounds including metabolites and alkaloids. Finally, a variety of metal binding signatures are found in sproteins suggesting their affinity for metal ions, mostly calcium, zinc and magnesium. These results strongly indicate that many novel small proteins are fully functional, playing roles in important cellular processes. Data collected here is freely available to the academic community at http://www.brylinski.org/content/databases; these resources can be used to assist targeted studies oriented on elucidating the functions of hypothetical small proteins.

## Methods

### Short protein sequences

In this study, we use sproteins identified in the FANTOM collection of mouse cDNAs [10] by Frith *et al*. [12]. From the original dataset, we selected 3,556 sequences 50–100 amino acids in length for structure modeling and the subsequent structure-based function annotation.

### Meta-threading and structure modeling

Full-length structure models of sprotein sequences are constructed using *e*Thread, a recently developed meta-threading pipeline for protein structure modeling [38,39]. *e*Thread integrates ten state-of-the-art single threading algorithms for the selection of template proteins from a non-redundant PDB library [59]: COMPASS [60], CS/CSI-BLAST [61], HHpred [62], HMMER [63], pfTools [64], pGenThreader [65], SAM-T2K [66], SPARKS [67], SP3 [67] and Threader [68]. All-atom models are built from meta-threading alignments using *e*Thread/Modeller, which employs a widely used template-based modeling package, Modeller [69]. Each model is assigned a confidence by *e*Rank/Modeller [39]. The resulting models are assessed in terms of the secondary structure content assigned by STRIDE [70], the hydrogen bond pattern calculated by HBPLUS [71], and the stereochemical quality inspected by PROCHECK [43].

### Structural classification

Confidently predicted models of sproteins are subject to structural classification. Here, we use a subset of the CATH Protein Structure Classification [37] library containing 22,374 representative protein domain structures, in which redundancy is removed at the 95% global sequence identity. Each sprotein model is structurally aligned to all CATH domains using Fr-TM-align program [72]; subsequently, CATH classification is transferred from the best structural hit. We note that Fr-TM-align employs TM-score structural similarity metric [40], which is protein length independent, ranges from 0 to 1 and has a well defined structural similarity threshold at 0.4.

### Modeling of protein-protein interactions

Putative interactions between sproteins and the remaining gene products in the mouse proteome are modeled using a template-based approach. As a template library, we use a representative and non-redundant at 40% sequence similarity dataset of experimentally solved protein dimers culled from PDB [58]. This library comprises 8,155 dimers, in which the monomers are 50–600 residues in length [36]. In each dimer, the shorter monomer is used as a template for sproteins and the longer is taken as its putative receptor. First, we identify protein binding residues in the modeled structures of sproteins using PINUP [44]. Next, each sprotein is structurally aligned onto all template structures in the dimer library using Fr-TM-align. For statistically significant structural hits at a TM-score of ≥0.4, we calculate Matthew’s correlation coefficient (MCC) between interfacial residues as found in the experimental template structure and putative binding residues predicted for the sprotein by PINUP. A template structure is used further only when MCC is ≥0.5, which indicates a substantial overlap.

Receptor proteins from the dimer library are mapped to the entire mouse proteome using sequence profile-profile comparisons. First, we construct a profile hidden Markov model (HMM) for each receptor and scan it through a set of HMMs built for 37,837 gene products 50-600aa in length from the mouse proteome. Here, we use the mouse assembly GRCm38.69 released by Ensembl [73] and pairwise alignments by HHsearch [62], which employs a sensitive method for detecting homologous relationships between proteins. Next, we keep only these mouse sequences that have a probability score calculated by HHsearch of >0.5, which suggests that they are likely to be related to the receptor also at the structural level. Finally, we mount each highly scored mouse sequence in the receptor structure according to the profile HMM-HMM alignment and evaluate the binding energy against the sprotein structurally aligned onto the template. Here, we use sequence-specific protein docking potentials (PDPs) [74], which provide an accurate measure for detecting protein-protein interactions. We also collect interaction energies for the parental crystal structures of complexes in the template library; these are used to assign *p*-values to the predicted interactions from the statistical distribution of PDP scores in known protein-protein complexes (fitting plots are shown in Additional file 2: Figure S2).

### Ligand-binding prediction

To annotate sproteins with ligand-binding sites, we use a recently developed *e*FindSite [46], which has improved prediction accuracy against protein models compared to its predecessor, FINDSITE [75]. *e*FindSite not only predicts binding sites and residues, but also constructs consensus molecular fingerprints of putative binding ligands. These are used to carry out ligand-based virtual screening in order to identify small organic compounds that likely bind to the interaction sites predicted in sproteins. We use two screening libraries: KEGG compound [52] that contains 11,265 molecules known to bind to protein targets and a non-redundant at a Tanimoto coefficient [76] of 0.8 ZINC12 [77] collection of 244,659 commercially available organic compounds.

### Metal-binding prediction

Metal binding sites and binding residues are predicted in sprotein models using FINDSITE-metal [54], which was demonstrated to be applicable in genome-wide projects. To further increase the accuracy and sensitivity of metal binding site detection, we replaced the original single threading template identification algorithm with meta-threading using *e*Thread as described in [36].

## Competing interests

The author declares that he has no competing interests.

## Supplementary Material

Additional file 1 **Structure quality assesment for sprotein models.** Correlation between TM-score estimated by *e*Thread and GDT-score estimated by APOLLO for structure models constructed for sprotein sequences from the mouse proteome. Click here for file

Additional file 2 **Distribution of PDP scores across experimental dimer structures.** Distribution of the Protein Docking Potential (PDP) score per residue for a non-redundant dataset of the crystal structures of protein-protein complexes. The probability density function and the cumulative distribution function is shown in **A** and **B**, respectively. In both graphs, Gaussian fit to the empirical data is shown as a black dashed line. Click here for file
