# Supplementary figures and images for: Exploring the “dark matter” of a mammalian proteome by protein structure and function modeling
Source: Proteome Sci. 2013 Dec 9;11:47. doi: 10.1186/1477-5956-11-47 (PMC3866606; doi:10.1186/1477-5956-11-47)

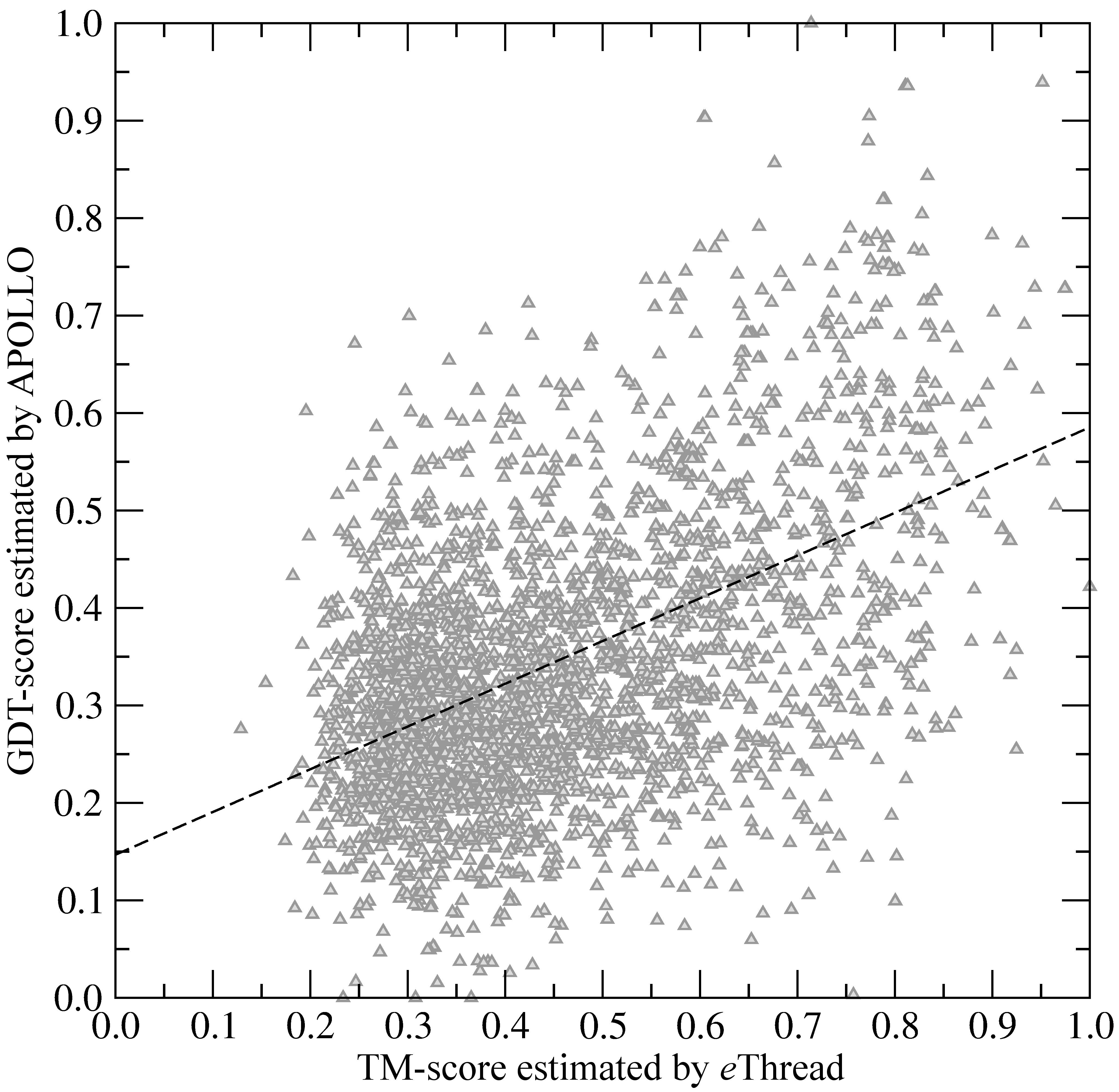

Supplement: Additional file 1 — Structure quality assesment for sprotein models. Correlation between TM-score estimated by eThread and GDT-score estimated by APOLLO for structure models constructed for sprotein sequences from the mouse proteome. [file 1477-5956-11-47-S1.tiff]

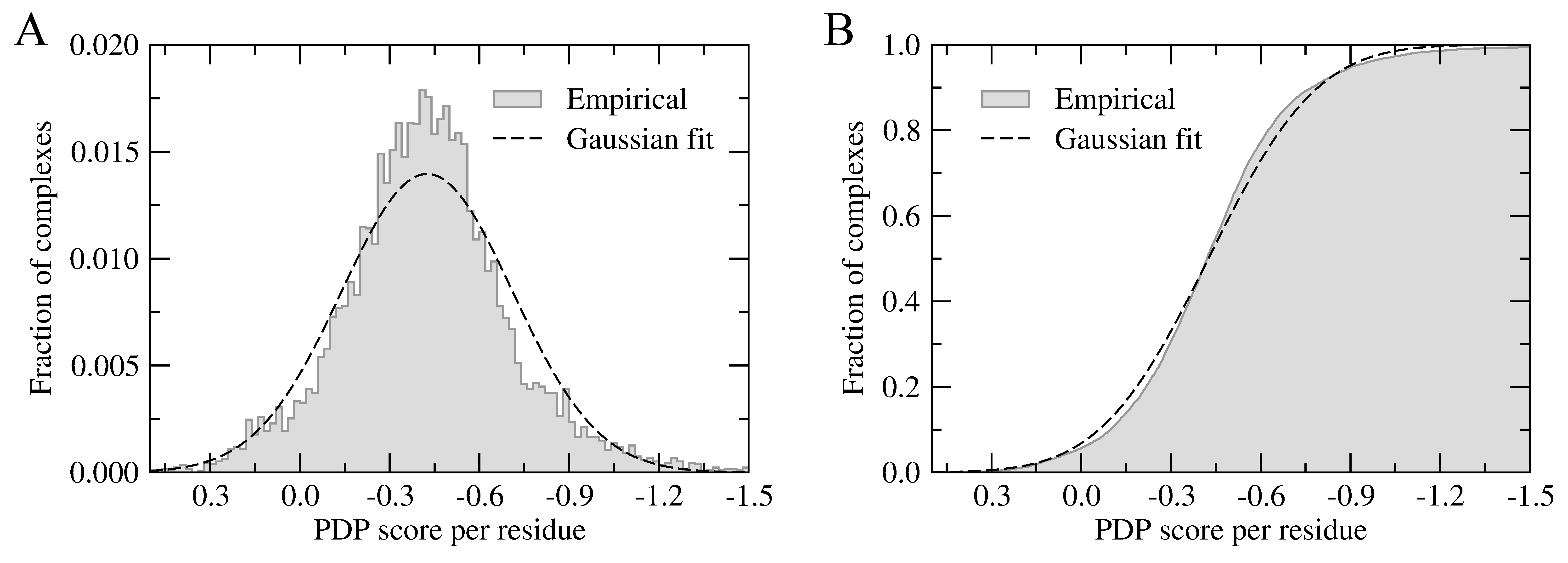

Supplement: Additional file 2 — Distribution of PDP scores across experimental dimer structures. Distribution of the Protein Docking Potential (PDP) score per residue for a non-redundant dataset of the crystal structures of protein-protein complexes. The probability density function and the cumulative distribution function is shown in A and B, respectively. In both graphs, Gaussian fit to the empirical data is shown as a black dashed line. [file 1477-5956-11-47-S2.tiff]
